# Useful Learning From Bachelor's Thesis to Professional Nursing Practice: A Qualitative Interview Study

**DOI:** 10.1177/23779608251317111

**Published:** 2025-01-29

**Authors:** Ani Henttonen, Marianne Teräs, Max Scheja, Bjöörn Fossum, Margareta Westerbotn

**Affiliations:** 1 Department of Health Promoting Science, 25548 Sophiahemmet University, Stockholm Sweden; 2 Department of Learning, Informatics, Management and Ethics, 27106 Karolinska Institutet, Stockholm, Sweden; 3 Department of Education, 7675 Stockholm University, Stockholm, Sweden; 4 Department of Nursing Science, 25548 Sophiahemmet University, Stockholm, Sweden

**Keywords:** bachelor’s education, professional development, nursing science

## Abstract

**Introduction:**

It is well-established in research that clinical learning during nursing education is a foundational preparation for future practice. However, the role of academic tasks, such as writing a bachelor's thesis, is less recognized for its contributions to nurses’ working lives and overall professional development.

**Objective:**

This study aimed to explore registered nurses’ perceptions of the process of bachelor's thesis and its perceived usefulness in professional nursing careers.

**Methods:**

Semistructured interviews with ten registered nurses was employed. A thematic analysis of the data and the framework of sustainable learning were used.

**Results:**

The findings showed that the process of writing a bachelor's thesis during nursing education was perceived as a valuable learning experience and a source of continuing development. The main themes, professional knowing and professional agency, demonstrate the knowing applied in the healthcare contexts and for understanding nurse-patient relationships. The nurses stated that professional agency in research dissemination and a critical approach in their daily patient care was important and that the bachelor's thesis had provided a foundation for these practices.

**Conclusion:**

Writing a bachelor's thesis during nursing education is justified as it may contribute to a critical understanding of nursing phenomena and the care taking place in clinical contexts. Bachelor's theses align with sustainable learning and are one of the prerequisites for readiness for change and development in professional practice.

## Introduction

A bachelor's thesis (BT) is regarded as a bench mark to evidence-based methods and quality in the practice of nursing care (Patelarou et al., 2020). Nursing students engage in academic reading and writing throughout their education, with the BT typically undertaken in their final year of study. However, despite this training, research utilization by nurses in clinical practice is shown to be low (Rudman et al., 2020). A recent study showed that due to workflow being unpredictable, clinical nurses have less opportunities to keep abreast of new knowledge in their specialty fields of work (Ramjan et al., 2022). Other reasons for low engagement regard professional boundaries obstructing creative discussions and implementation of nursing research at healthcare working places (Clarke et al., 2021). Since BTs are shown to be an important part of academic socialization processes, it is of interest to investigate their usefulness to registered nurses in their working life.

## Review of Literature

Academic education of nurses in Sweden is built on the European model of first cycle higher education (Swedish Council for Higher Education, 2020). The education to registered nurses therefore equals to studies at bachelor's level. Typical for the level of bachelor's is the final academic BT, which many students see as a final proof of their becoming nurses (Lundgren & Halvarsson, 2009; Mitchell et al., 2020). The academic expectations that follow with the task have not been a long tradition among the practical professions’ education (Strauss, 2017); in Sweden, BTs in nursing science have been written as mandatory examinations since 2007. Students are shown to be devoted to knowing more in their own field and to diving deep into topics that are relevant to their professional life as nurses (Henttonen et al., 2021). Thus, the bridge from university context to working life context is seemingly obvious; however, not much is known about the linkage from the perspective of registered nurses who currently work in healthcare.

Reports from branches other than healthcare emphasize the importance of academic communication in visualizing, analyzing, and justifying the connections between science, theory, and practice in professional working situations (Horning et al., 2019; Zou et al., 2022). Indeed, a condition for a deeper knowing in practice is that members of a profession, such as nursing, engage in theorizing about their work (Gherardi, 2012).

Nursing education that includes a written thesis and previous research experiences are found to enhance positive attitudes to research (Gros-navés et al., 2022; Grønning et al., 2022). At work, healthcare staffs are encouraged to make research-based decisions (Clarke et al., 2021). In maintaining research skills, local and group-based journal clubs are previously shown to be a fruitful activity to discuss, appraise, and inform about research (Friesen-Storms et al., 2015; Mattila et al., 2013). The role of a leader has been found to be of great importance enhancing and maintaining work-based knowledge and research practices (Lunden et al., 2017). Based on the previous literature presented in this matter, we assert that if the knowledge and skills gained from research training, such as writing an academic BT, are not used, they can be seen as knowledge waste and may present an obstacle to the integration of theoretical knowledge and research. This study focuses on registered nurses who currently work and who had written a BT during their nursing education.

## Theoretical Framework

The study is grounded in the framework of sustainable learning, denoted as an individual's agency to learn throughout life (Ben-Eliyahu, 2021). The concept of sustainable learning is an umbrella term, including aspects of continuing and lifelong learning (Mlambo et al., 2021), transferable knowledge (Aita et al., 2007; Lundgren & Robertsson, 2013), research utilization (Rudman et al., 2020), and development and self-regulation (Anåker & Elf, 2014; Brydges & Butler, 2012). Writing a BT involves skills in literacy and science; however, these are also highlighted among the key competences in the EU framework for 21st-century working life (European Union, 2018). Thus, sustainable learning leads to outcomes that are broad and thus encompass both professional and personal development: “When acquiring SLE [Sustainable learning in education], the learner becomes self-sufficient, can self-evaluate their learning and its usefulness, adjust the learning content or process to align with their needs and aims” (Ben-Eliyahu, 2021, p. 3). In this way, applying the principles of sustainable learning in concrete ways within professional nursing aligns with a holistic understanding of human health conditions and promotes a judged use of products and services in patient care (Anåker & Elf, 2014).

## Materials and Methods

The research was designed as a qualitative semi-structured interview study, based on the following research question: What are clinical nurses’ perceptions of the influence of writing a BT during their education on their professional working life practices?

We applied thematic analysis (TA) with six steps of interpretation to the data (Braun & Clarke, 2021). COREQ protocol was used in reporting the study (Tong et al., 2007).

## Participants and Data Collection

A purposeful sample of participants was recruited through an alumni network of former students in one university in Sweden. The criteria for participant eligibility were, firstly, having completed a BT during nursing education and secondly and having worked at least 1 year after graduation. Invitation emails were sent in September 2022 and a reminder at week 2. A total of 10 interviews, with 6 females and 4 males, were carried out using semistructured interview guide (Kvale, 2007). The interview guide is available as Supplemental material. The participants were between 29 and 61 years of age, representing a range of 1–17 years in profession as registered nurses with a bachelor's degree. They currently worked in gynecology, hemodialysis, surgery, cardiology, and psychiatric care.

Data collection with individual interviews took place from October 2022 to February 2023 by the first author. Due to the preferences of the participants, the interviews were carried out by a digital video conferencing platform. A separate voice recorder was used to document the audio of the interviews only. Prior to the interviews, the participants were informed about the research goals, methods, and their rights as informants emphasizing the voluntary nature of participation. Verbal informed consent was obtained from all the participants.

The interviews lasted 20–60 min. The semistructure interview questions allowed for an open discussion from the part of the participant leading to not all the interview questions necessarily became posed. The first interview as a pilot was included in the study due to no changes in the interview guide (Polit & Beck, 2021).

## Analysis

TA with an inductive approach, described by Braun and Clarke (Braun & Clarke, 2021, 2006), was used. TA is a method for identifying, analyzing, and reporting themes (patterns) within data and commonly presented in six phases of analysis: familiarizing with data, generating initial codes, searching for themes, reviewing themes, defining and naming themes, and producing the report. The data were transcribed and read iteratively and multiple times for familiarization. In the next phase, the data were coded by identifying phrases and words from the transcripts that corresponded to the study objective. Along the analysis process, connections between the thesis experience and the nurses’ current work were identified in the data and coded. The analysis continued by identifying sets of subthemes and themes, which were organized based on their conceptual differences and similarities. For example, relationships were identified in the data when participants described how the thesis work had been useful and had “opened many doors” in terms of the current working life. These features in the data were discussed as indicators of long-term growth and adaptability as nursing professionals. In the final phase of analysis, the framework of sustainable learning was found to confirm the relevance and meaning of the findings. In this way, final themes were drawn from the mapped, reviewed, and refined subthemes (Braun & Clarke, 2006). The first author (AH) led the analysis process including naming the final themes. The last author (MW) reviewed each stage of the analysis process and participated in the process of coding. The content was discussed in the research team and agreement was reached regarding the analysis, as suggested by Nowell et al. (2017). Quotations were chosen to characterize the themes. Two main themes and subthemes are visualized in Table 1.

**Table 1.** Generated Subthemes and Main Themes.

Main themes	Subthemes
Professional knowing	Applying of findings from the BT to patient care
	Viewing patients as subjects
	Identifying gaps of knowing
Professional agency	Research dissemination
	Critical approach
	Creativeness

## Research Ethics and Consent to Participate

The Declaration of Helsinki is adhered to in this study (WMA, 2013). Furthermore, guidelines for the ethical assessment of research involving humans have been adhered to throughout the research process (Swedish Research Council, 2017). Participants were provided with information detailing the study's purpose, the voluntary nature of participation, and the right to withdraw at any time without consequence. Informed consent was obtained from all the participants prior to the interviews. Additionally, confidentiality was maintained by using coded identifiers for email addresses and names. The information and the original recordings were kept separate in a password-protected computer program, accessible only to the first author and will be destroyed after publication. The study has obtained an approval from the Ethical Review Board, Stockholm (2017/639-31/5).

## Results

The results reveal two main themes, professional knowing and professional agency, each encompassing three subthemes as demonstrated in Table 1.

The results, along with the selected citations from the interviews, are elaborated in the following text.

## Professional Knowing

The theme professional knowing refers to concrete care interventions in *applying findings from the BT to patient care*. *Viewing patients as subjects* signifies an understanding of their patients as equals and the significance of nursing care to them. Moreover, writing a BT had the influence of facilitating the identification of gaps in their professional knowing*.*

### Applying Findings From the BT to Patient Care

Most nurses perceived significant professional use from their BT, allowing them to apply their findings. The process of writing a BT had informed their patient care, and the nurses described curious efforts to apply this knowledge in their work situations:

Yes, it [the BT] probably contributed to my dedication [sleep promotion] since I thought it was important…the light bulbs…and I tried to create a better situation and question how fit for purpose the patients’ beds were. (Nurse no. 3)

In this case, the testing of findings from a BT was conducted without involvement from colleagues and management.

### Viewing Patients as Subjects

The process of writing a BT had included reading and writing about principles of humanity and justice in nursing care. This process convinced them of the ethical and philosophical principles where a patient is considered as a subject, emphasizing that care relations should be based on equality between humans. The BT had demonstrated what might be crucial for individual patients and should be prioritized in nursing care. Insights applicable to professional knowing were also gained, as expressed by one nurse:

I don’t think the topic itself directly helped me in my professional work. Though I have thought about the BT over the years, so irrespective of whether a person has HIV or not, that how patients experience our care. I mean the question of care-encounters have been important to me throughout the whole of my nursing career. I mean the professionality, how I as a nurse can impact the first encounter so that it [the BT] has given me something, I have to say. (Nurse no. 7)

The participants unanimously expressed that the experience of writing a BT on patients’ experiences of healthcare and treatments had afforded them valuable understanding of potential patient responses and reactions to their care interventions.

### Identifying Gaps of Knowing

A majority of the nurses were not employed within specialties they once had written their BT about. Nevertheless, they reflected that the BT had facilitated the identification of gaps in their current professional knowing. The realization of these knowledge deficits and the lack of existing research prompts them to use literature screening skills acquired during the BT process. Furthermore, these knowledge gaps stimulate discussions with colleagues and other health professionals in the workplace:

I have absolutely done that [searched literature] when questions have popped up what there is no evidence for. For example, there is a lot of discussions among registered nurses whether to wear a cap or not in the operation theatre. I have learnt, research is lacking in many areas. (Nurse no. 2)

Discussions at workplace were seen important as the nurses perceived that they had lacked an understanding of the difference between academic and clinical professional knowing at the time of writing their BT.

## Professional Agency

Overall, the participating nurses articulated that the process of writing a BT had endowed them with the readiness to act on their knowledge in practical scenarios. Opportunities to act on professional knowledge could manifest through research dissemination, critical approach, and creativeness.

### Research Dissemination

The nurses reflected that having written a BT had facilitated identification of avenues for collaboration with professionals across a range of contexts. Also had the BT process prepared them to draw from and disseminate scientific knowledge in various contexts.

The BT has given me unbelievable tremendous benefits, it's opened very many doors to me, regarding my work and now in my position as a nursing leader. I have acquired a lot of knowledge from the BT that I can refer to when we discuss things here [the clinical ward]. I can then say that this is not something that I have come up with myself, but it is from research… It actually led me to giving lectures for university students, for staff at medical units and for social services personnel. (Nurse no. 6)

The experience of the process of BT on diverse topics and disseminating scientific research acted as a catalyst for the development of their professional networks.

### Critical Approach

The participating nurses reported that having written a BT had equipped them with the ability to discern scientific knowledge from general and potentially erroneous opinions in the context of healthcare. One nurse expressed it in the following manner:

Yes, I absolutely have had benefits from having written a BT. It is rather nice to be able to lean towards evidence, that it is not only to have opinions about things. I actually have done a BT where it showed that ones’ own stress or bad mood can affect how one assesses patients. So, these things impact and are transferred to care. It is easier to reflect on such things when you’ve done a study yourself. It was a fascinating exercise like a detective work and I think it probably impacts on how I thereafter think about various things. (Nurse no. 10)

In this way, having written a BT had given the participant nurses a ground for critical approach to reflect on the components of professional patient care.

### Creativeness

The participating nurses reflected on the obvious relationships between the theoretical frameworks in nursing explored in their BTs and their professional endeavors. They found that these relationships enabled them to establish new connections to the application of scientific and theoretical knowing in their professional contexts.

I find a clear relationship between nursing science and theory in the psychiatric care compared to the somatic care. So, the existing nursing theories, which I already had recognized, are very applicable and so, it was good to investigate [in a BT] which safety factors would actually be the most important ones. (Nurse no. 4)

Writing a BT had provided the nurses with an understanding of the interplay between scientific inquiry and theoretical constructs. Consequently, in their professional lives, familiarity with these theories facilitated and inspired the generation of new ideas and connections to theoretical concepts. In this way, an ongoing and creative dialogue between research and professional everyday nursing is maintained.

## Discussion

This study investigated the perceptions of the usefulness of having written a BT among registered nurses who have been working in the profession for between 1 and 17 years. In the following, we discuss the challenges and opportunities of bringing educational and professional value to BTs, based on our findings. Since not all educational experiences are clearly aligned with the demands of the profession, the discussion highlights BTs as key approach in bridging the gap between learning in higher education and ongoing professional development among registered nurses. Additionally, we propose more concrete educational and practical implications, incorporating both academic and professional knowing and agency. By doing so, we believe that nursing professionals can be supported in their efforts of learning throughout their careers.

## Professional Knowing and Agency

The results show that the registered nurses acknowledged that the minor research task involving academic writing of a BT still influenced their professional practice. The process of writing a BT had helped the nurses to establish and enhance their professional knowing and agency in their working practices. This finding is surprising, considering that the BT was completed up to 17 years earlier. While both main themes show the registered nurses’ current professionalism in working life activities, they mirror the process of writing a BT pursued during their nursing education. This study highlights that common to both professional knowing and professional agency is an ongoing reflective development of knowledge and skills.

Based on the main themes, professional knowing and professional agency, the results highlight the nurses’ perspective on a lifelong and sustainable learning taking place in working life.

Our results confirm the concepts of professional knowing and agency comprising several components. Previously, Gherardi (2012) identified two fundamental aspects of professional knowing: the reproduction of existing knowledge and the creation of new knowledge objects.

Professional agency refers to active engagement and authority in the professional field (Eteläpelto et al., 2013). Our findings suggest that this may also contribute to forging new connections between nursing theory and clinical nursing. Therefore, professional agency encompasses conscious decisions and reflections even when knowledge is being routinely reproduced. According to Eteläpelto et al. (2013), the concept of agency relates to both individual and social dimensions (Eteläpelto et al., 2013). This suggests that in the context of BT, the nurses’ professional work in this study encompasses both individual and social activities. The ability, identified in this study as professional agency, highlights the nurses’ seizing opportunities in patient care, among colleagues and within communities, which is stated as important for effecting change and enhancing quality of care (Kipinä et al., 2024).

## Sustainable Learning From Bachelor's Theses in Nursing Education

Previous studies show that during their education, students may not always appreciate the significance of undertaking a BT to their future professional practice (Johansson & Silén, 2018; Mattsson, 2016). Zou et al. (2022) asserted that undergraduates preferred research to be anchored to real-life problems rather than to research only, showing a preference for a professional identity, rather than research identity. This highlights a prevailing research-practice divide, often criticized as an academic construct that primarily serves those pursuing scientific careers (Aguayo-González & Leyva-Moral, 2019). In contrast, majority of the nurses in this study expressed the importance of having completed their BTs, indicating that nursing practice has evolved beyond its practical knowledge domains, also confirmed in later studies (Fernández-Cano et al., 2021; Gros-navés et al., 2022). However, the results in this study also point toward a continuing need of educational interventions that promote students’ academic and theoretical approach to nursing. A recent review highlights that explicit pedagogies which integrate theoretical knowledge and science into clinical practice are rare (Westerdahl et al., 2022). Consequently, the skills and knowledge assessed in a BT may often be evaluated independently of clinical learning, both in context and timing. Reciprocally, the national clinical examination, conducted in the final year of nursing studies, may be constructed to assess nurses’ clinical reasoning and performance primarily from a procedural skills perspective (Lilja Andersson et al., 2013).

## Usefulness of Bachelor's Theses to the Development of Professional Knowing and Agency

In terms of lifelong learning, professional nurses are expected to maintain their individual interest in scientific information and healthcare professionals generally have a responsibility to stay updated with the latest evidence. However, a Swedish study indicated nurses only engaged in evidence-based care practices occasionally, approximately twice a year (Rudman et al., 2020). Furthermore, while the use of both nursing theory and research is claimed to reduce random and unfinished care, they are often juxtaposed as either opposing or supporting the automatization and standardization of nursing practice (Younas & Quennell, 2019). Nevertheless, our study confirms previous research on the perceived value of continuing professional development (Mlambo et al., 2021), as the nurses were aware of and actively assessed deficiencies in their theoretical and scientific knowing. Research knowledge is known to have a high perishability. Therefore, it is important to highlight the professional utility of a BT, as it is probably the only hands-on research activity engaged in during nursing education. However, based on this study, we maintain that professional knowing and agency involve evidence dissemination across organizational and practice boundaries and professional roles, as also found by Wollscheid and Opheim (2016).

Our findings support the idea of nurses’ work as a cognitive interplay between theory and practice (Benner et al., 2009). Our findings also corroborate earlier studies on the transfer and application of knowledge from nursing education to professional work life (Lauder et al., 1999; Lundgren & Robertsson, 2013).

Therefore, a knowledge culture is needed where agentive nurses actively monitor and engage with research produced for the discipline (Jensen, 2007).

The findings in this study reinforce the concept of a BT as a tool for accessing knowledge that is specific for nursing (Henttonen et al., 2023; Markauskaite & Goodyear, 2017).

## Educational and Practical Implications

It is indicated in this study that the skills and knowledge gained need continuous implementation and updates. Therefore, we propose three educational and practical implications: Firstly, students should be trained to integrate research into their clinical decision making during any clinical examination. This would exercise research communication and students’ skills to formulate explanations and motives of nursing interventions as well as evidence on patient responses to the nursing care provided. Secondly, registered nurses acting as clinical supervisors could encourage students to search for and report on research relevant to patient care in the ward. This initiative would facilitate the dissemination and discussion of valuable nursing knowledge, encompassing both nurses’ and patients’ perspectives on healthcare provision.

We maintain that nursing science is a specialized niche within the field of healthcare, characterized by nurses’ dedication to patients’ health and well-being (Benner et al., 2009). In healthcare organizations, where library services are available, these units cater to medical information needs and educate personnel on search systems. Based on our findings, our third proposal is that nursing staff would benefit from tailored library services designed to meet their specific information needs. This approach would promote inquiry-based workplace learning (Baek et al., 2023).

The study underlies the importance of sustainable learning. Unlike rigid protocols, we emphasize the value of a BT. Given the ongoing digital revolution in society, there is a risk of relegating the BT solely to an academic exercise. A concern for the future is that tasks, like writing a BT, may not be prioritized in nursing education unless the utility of a BT is more strongly emphasized.

Our results indicate that a BT can contribute to knowledge practices in nursing that equals to sustainable learning. However, while standard competency assessments typically focus on quantifiable skills, there is a paucity of research on nurses’ perspectives regarding the ongoing application of knowledge and skills acquired through a BT. Therefore, further research is warranted to establish and sustain these connections.

## Strengths and Limitations

There are potential methodological limitations to the present study that should be acknowledged. Our research objective entailed a dual focus: recalling a specific task from the nurses’ former education and assessing their perceptions of its usefulness in their current professional practice. Although the undergraduate thesis work was emphasized as the interview context, it is possible that professional development results from multiple influences. Additionally, some nurses had completed master's theses or other written projects since their bachelor's education, which could pose a threat to the study's trustworthiness. The TA employed to analyze the data was not intended to cover the entire spectrum of phenomena, but rather to identify patterns relevant to the study's aim. A total of 10 registered nurses participated, which may not guarantee a coverage of plausible important aspects related to the phenomenon.

Malterud et al. (2016) propose the concept of information power to guide adequate sample sizes in qualitative studies (Malterud et al., 2016). To address a balance and bolster the study's dependability, representative quotations were meticulously and collaboratively selected by the authors. The nurses interviewed in this study had varied experiences in writing their BTs, ranging from minor empirical investigations to literature reviews. This diversity is congruent with the differing local guidelines and practices for BT writing stipulated by universities. Moreover, the nurses worked in various fields of healthcare, potentially enhancing the transferability of the results.

## Conclusions

The study concludes that writing a BT during nursing education imparts learning qualities that can be transferred and developed to professional knowing and agency in the workplace. In the intricate and knowledge-intensive field of healthcare, having written a BT serves as an independent framework that reinforces nurses’ identities as professional experts. Consequently, a BT may establish a basis for sustainable learning, enabling nurses to critically evaluate the applicability of standard healthcare and crucially, the quality of patient care provided.

## Supplemental Material

sj-docx-1-son-10.1177_23779608251317111 - Supplemental material for Useful Learning From Bachelor's Thesis to Professional Nursing Practice: A Qualitative Interview Study Supplemental material, sj-docx-1-son-10.1177_23779608251317111 for Useful Learning From Bachelor's Thesis to Professional Nursing Practice: A Qualitative Interview Study by Ani Henttonen, Marianne Teräs, Max Scheja, Bjöörn Fossum and Margareta Westerbotn in SAGE Open Nursing

sj-docx-2-son-10.1177_23779608251317111 - Supplemental material for Useful Learning From Bachelor's Thesis to Professional Nursing Practice: A Qualitative Interview Study Supplemental material, sj-docx-2-son-10.1177_23779608251317111 for Useful Learning From Bachelor's Thesis to Professional Nursing Practice: A Qualitative Interview Study by Ani Henttonen, Marianne Teräs, Max Scheja, Bjöörn Fossum and Margareta Westerbotn in SAGE Open Nursing
