# Supplementary material for: Useful Learning From Bachelor's Thesis to Professional Nursing Practice: A Qualitative Interview Study
Source: SAGE Open Nurs. 2025 Jan 29;11:23779608251317111. doi: 10.1177/23779608251317111 (PMC11775977; doi:10.1177/23779608251317111)
Supplement: sj-docx-2-son-10.1177_23779608251317111 - Supplemental material for Useful Learning From Bachelor's Thesis to Professional Nursing Practice: A Qualitative Interview Study [file sj-docx-2-son-10.1177_23779608251317111.docx]

**Interview guide**

**Opening question**

**-**What are your thoughts about having written a Bachelor’s thesis during your first-cycle nursing program?

- What was the topic of your Bachelor’s thesis?

- How was the writing process at the time? Were there any surprises?

- What do you consider is the most important thing you learnt by writing a Bachelor’s thesis?

**Questions relating to the profession and working life**

- What are your thoughts on the usefulness of writing a Bachelor’s thesis during nursing education?

-What are your thoughts about the process of thesis’ work in relation to your professional work?

-Thinking back, are there any key takeaways from the Bachelor’s thesis to your work as a nursing professional?

- Is there any other topic that you might have considered or currently would need to know more about to support you in your professional work?

**Closing** **question**

-Is there anything else you would like to add regarding the topics we have covered through this interview and our discussion?
